# Impact of C-reactive protein–triglyceride–glucose and systemic immune-inflammation indices on obstructive sleep apnea in older adults with depression

**DOI:** 10.3389/fnins.2026.1821053

**Published:** 2026-06-17

**Authors:** Shujing Hu, Jinxuan Zheng, Shunyao Xu, Zhiyuan Lin, Shanshan Zhu

**Affiliations:** Department of Psychiatry, Wenzhou Seventh Peoples Hospital, Wenzhou, China

**Keywords:** C-reactive protein-triglyceride glucose index, depression, obstructive sleep apnea, older adult, systemic immune-inflammation index

## Abstract

**Objective:**

Both the C-reactive protein (CRP)–triglyceride–glucose index (CTI) and the systemic immune-inflammation index (SII) are easily accessible, cost-effective, and rapid indices derived from biochemical examinations. The study aimed to identify the roles of the CTI and SII in older adults with comorbid obstructive sleep apnea (OSA) and depression.

**Methods:**

The study included 52 older patients with depression coexisting with OSA and 108 patients with depression but without OSA. The CTI was calculated using the following equation: 0.412 × Ln (CRP) (mg/dL) + Ln [triglyceride (mg/dL) × fasting glucose (mg/dL)/2]. The SII was calculated using the formula: platelet × neutrophil/lymphocyte.

**Results:**

A greater proportion of men and a higher mean body mass index were found in older adults with comorbid OSA and depression compared to those with depression only (*p* < 0.05). Older adults with comorbid OSA and depression also showed higher levels of fasting glucose, triglycerides, CRP, the triglyceride–glucose (TyG) index, and the CTI than those with depression alone. We also found that older adults with comorbid OSA and depression had higher neutrophil counts, a higher neutrophil-to-lymphocyte ratio (NLR), and a higher platelet-to-lymphocyte ratio (PLR), and an increased SII compared to those with depression alone. Logistic regression analysis demonstrated that male sex and higher CTI and SII values were correlated with the presence of OSA in patients with depression.

**Conclusion:**

The study demonstrated that higher CTI and SII values may be associated with comorbid OSA and depression in older adults.

## Introduction

Depression is one of the most common mental disorders among older adults, affecting almost every sphere of their lives (Das et al., 2025). An increasing proportion of the aging population has made depression in older adults a growing public health concern worldwide (Zhang et al., 2025). Despite the complex etiology and the comorbidity of somatic diseases lead to underdiagnosis of depressive disorders in this cohort, approximately 10% of older adults still experience major depressive disorders globally (Verhaak et al., 2014; Li et al., 2025). Obstructive sleep apnea (OSA) is a common chronic sleep-related breathing disorder in the older adult population, and epidemiological data indicate that OSA affects up to 36.3% of patients with major depressive disorder (Bao et al., 2017; Stubbs et al., 2016). Conversely, the presence of OSA is associated with an increased risk of developing depression. This is because the white matter changes resulting from hypoxemia and sleep fragmentation can impair key cognitive functions such as attention and memory (Kao et al., 2025). Therefore, there may be a complex, bidirectional relationship between depression and OSA, which has led to an increasing interest in elucidating the shared pathophysiological mechanisms between the two conditions (Liao et al., 2025). These mechanisms include hypoxemia-mediated neuropathology (Zhu et al., 2025), inflammatory dysregulation (Fiedorczuk et al., 2023; Altamura et al., 2025), dysfunction in shared pathways for airway control and mood regulation (Lu et al., 2025), and common comorbidities such as cardiovascular disease, diabetes, obesity, and hypertension (Zhou et al., 2024; Melaku et al., 2024; Marquina et al., 2023).

Both the C-reactive protein (CRP)–triglyceride–glucose index (CTI) and systemic immune-inflammation index (SII) are easily accessible, cost-effective, and rapid indices derived from biochemical examinations, which have exhibited clinical significance in various diseases affecting the older adult population (Sun et al., 2025; Fan et al., 2025). The CTI integrates both inflammatory and metabolic factors to evaluate systemic inflammation and metabolic disturbances simultaneously (Ruan et al., 2022). An increased CTI value may contribute to a higher risk of depressive symptoms and OSA (Huang et al., 2024; Yang and Su, 2025). The SII has recently been investigated as a novel inflammatory marker, providing additional diagnostic and prognostic insights for many inflammatory conditions, including depression and OSA (Jung et al., 2026; Gunes and Gunaydin, 2024). However, limited evidence exists regarding the clinical value of the CTI and SII in patients with depression who also have comorbid OSA. The study analyzed and compared the CTI and SII between older patients who have depression along with OSA and those who have depression without OSA.

## Methods

### Study populations

The study included 52 older patients who had depression coexisting with OSA and 108 patients who had depression without OSA at Wenzhou Seventh People’s Hospital between February 2025 and December 2025. The inclusion criteria for patients with depression were as follows: age ≥ 65 years, a new diagnosis of depression based on the diagnostic criteria of the International Classification of Diseases, 10th edition (ICD-10), and a 24-item Hamilton Depression Rating Scale (HDRS-24) score of ≥ 8. Other inclusion criteria for patients with comorbid OSA and depression were a new diagnosis of OSA based on the Apnea–Hypopnea Index (AHI) (≥ 5/h) and excessive daytime sleepiness. Excessive daytime sleepiness was assessed using the Epworth sleepiness scale and defined as a final score >10 (Johns, 1991). Excessive daytime sleepiness is a hallmark symptom of OSA and was used as an inclusion criterion due to the older age of the study population (Ulander et al., 2022; Guo et al., 2020). The exclusion criteria included the presence of other psychiatric disorders, such as bipolar disorder, anxiety disorders, schizophrenia, and substance use disorders; neurological disorders; inflammatory or infectious diseases; chronic pulmonary disease; a history of substance abuse; or refusal to participate in this study. All participants provided written informed consent. The study was performed in strict accordance with the Declaration of Helsinki and received ethical approval from the Ethics Committee of Wenzhou Seventh People’s Hospital.

### Calculation of the CTI and SII

Before polysomnography (PSG), fasting blood samples were collected from treatment-naive, newly diagnosed patients with depression in the morning to obtain biochemical data. The CTI was calculated using the following equation: CTI = 0.412 × Ln (CRP) (mg/dL) + Ln [triglyceride (mg/dL) × fasting glucose (mg/dL)/2]. The levels of CRP, triglycerides, and fasting glucose were measured using the latex-enhanced immunoturbidimetric assay, enzymatic colorimetric method, and oxygen rate method, respectively, with a fully automated biochemical analyzer, Beckman AU5800 (Beckman Coulter, USA). The SII integrated platelets, neutrophils, and lymphocytes and was calculated using the following formula: platelet×neutrophil/lymphocyte. Platelet, neutrophil, and lymphocyte counts were measured using the Mindray BC-6800 automated hematology analyzer (Shenzhen, China) and reported as 10^9^ cells/L.

### Overnight polysomnography

Participants were asked to stay two consecutive nights in our hospital’s sleep laboratory, including a first night for habituation and a second night for complete PSG. According to participants’ usual bedtime, PSG was initiated between 22:00 and 24:00 and ended between 6:00 and 8:00. Complete PSG was monitored using the SOMNOmedics V6 Sleep System (SOMNOmedics, Germany) with a standard montage including a 6-channel electroencephalogram (F4-M1, F3-M2, C4-M1, C3-M2, O2-M1, and O1-M2), 2-channel electro-oculogram (E1-M2 and E2-M2), 2-channel chin electromyogram (chin1-chinZ and chin2-chinZ), leg electromyogram, electrocardiogram, thermal flow sensors at the nose and mouth, pressure sensors at the nose, inductive plethysmography bands at the chest and abdomen, microphone sensors for expiratory snoring events, a finger oximeter for arterial oxygen saturation, and body position sensors. Apnea was defined as a continuous cessation of airflow for ≥10 s, and hypopnea was defined as a ≥ 30% reduction in airflow for ≥10 s accompanied by a ≥ 3% reduction in oxygen saturation or electroencephalogram arousal from sleep, according to the American Academy of Sleep Medicine (AASM) guidelines (Berry et al., 2012). The AHI corresponds to the number of apneic and hypopneic episodes divided by total sleep time in hours. Sleep architecture for each patient was analyzed, including total sleep time (TST); sleep efficiency (SE); sleep latency (SL); wake after sleep onset (WASO); arousal index (ArI); rapid eye movement (REM) sleep; REM/TST (REM%); non-rapid eye movement stage 1 (N1), N1/TST (N1%); non-rapid eye movement stage 2 (N2), N2/TST (N2%); and non-rapid eye movement stage 3 (N3), N3/TST (N3%).

### Psychiatric assessments

The Chinese-language version of the HDRS-24 was used to assess depression severity in older patients (Zhu, 1985; Hamilton, 1960). The HDRS-24 consists of 24 items with Likert-scale responses of either 0–4 for 14 items or 0–2 for 10 items. The total score can range from 0 to 76, with higher scores indicating greater severity of depressive symptoms. The Cronbach’s *α* coefficient for the HDRS-24 in this study was 0.843.

### Statistical analysis

The normality of the distribution of continuous variables was evaluated using the Shapiro–Wilk test. Descriptive statistics were used to summarize continuous variables. When normal distribution was confirmed, continuous variables were presented as mean ± standard deviation (s.d.), and group differences were analyzed using the independent samples *t*-test. When variables were not normally distributed, they were presented as median with interquartile range (IQR), and group differences were analyzed using the Mann–Whitney U test. Categorical variables were reported as numbers and percentages, and group differences were analyzed using the chi-squared test and Fisher’s exact test. Logistic regression analysis was performed to identify factors significantly associated with the presence of OSA in older adults with depression. A significance level of less than 5% (*p* < 0.05) was set for all statistical tests. All statistical analyses were performed using SPSS Statistics (Version 22.0, IBM Corp., Armonk, NY, USA).

## Results

### Demographic and anthropometric characteristics of patients

**Figure 1:**
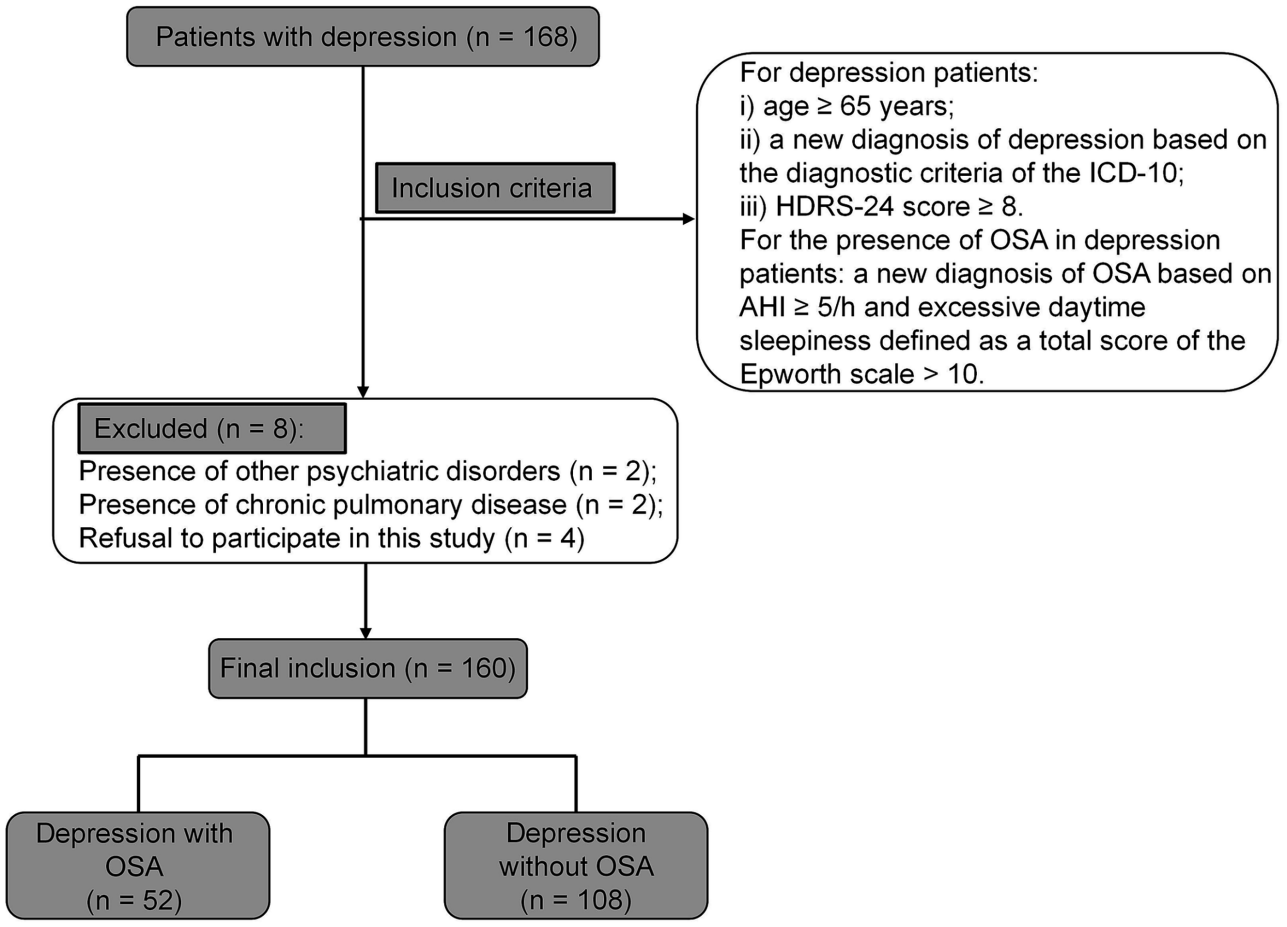
Flowchart illustrating patient selection and inclusion and exclusion criteria.

The study included 52 older patients with depression coexisting with OSA (AHI ≥ 5) and 108 with depression without OSA (AHI < 5) (Figure 1). Table 1 provides a summary of the patients’ demographic and clinical characteristics. A greater proportion of men and a higher mean body mass index (BMI) were found in older adults with comorbid OSA and depression than in those with depression only (*p* < 0.05). No significant differences were observed in age, current smoking status, alcohol use, education level, marital status, hypertension, diabetes, dyslipidemia, coronary heart disease, and antidepressant use between the two groups (*p* > 0.05).

**Table 1:** Demographic, anthropometric, and psychiatric characteristics of older patients with depression according to the presence of OSA.

Characteristic	Depression with OSA (*n* = 52)	Depression without OSA (*n* = 108)	*p*
Age (year), mean ± s.d.	73.0 ± 3.4	72.3 ± 2,7	0.182
Sex, *n* (%)			< 0.001
Male	38 (73.1%)	46 (42.6%)	
Female	14 (26.9%)	62 (57.4%)	
BMI (kg/m^2^), mean ± s.d.	25.1 ± 4.2	23.4 ± 3.7	0.008
Current smoking, *n* (%)			0.284
Yes	21 (40.4%)	33 (30.6%)	
No	31 (59.6%)	75 (69.4%)	
Alcohol use, *n* (%)	36 (69.2%)	63 (58.3%)	0.184
Educational level, *n* (%)			0.436
Middle school or less	36 (69.2%)	82 (75.9%)	
High school	15 (28.8%)	22 (20.4%)	
College or more	1 (2.0%)	4 (3.7%)	
Marital status, *n* (%)			0.943
Married	40 (76.9%)	81 (75.0%)	
Single, divorced, or separated	4 (7.7%)	10 (9.3%)	
Widowed	8 (15.4%)	17 (15.7%)	
Hypertension, *n* (%)			0.573
Yes	6 (11.1%)	16 (14.8%)	
No	46 (88.9%)	92 (85.2%)	
Diabetes, *n* (%)			0.095
Yes	8 (15.4%)	30 (27.4%)	
No	44 (84.6%)	76 (72.6%)	
Dyslipidemia, *n* (%)			0.544
Yes	1 (1.9%)	4 (3.7%)	
No	51 (98.1%)	104 (96.3%)	
Coronary heart disease, *n* (%)			0.323
Yes	0 (0.0%)	2 (1.9%)	
No	52 (100.0%)	106 (98.2%)	
Antidepressant use, *n* (%)			0.777
Yes	11 (21.2%)	25 (23.2%)	
No	41 (78.8%)	83 (76.8%)	

AHI, Apnea–Hypopnea Index; BMI, body mass index. Data summarized as mean ± s.d. were analyzed using the independent samples t-test. Count data were analyzed using the chi-squared test.

### Psychiatric characteristics and sleep architecture of older patients with depression according to the presence of OSA

As shown in Table 2, no significant differences were observed in the HAMD-24 scores between older adults with comorbid OSA and depression and those with depression only (*p* > 0.05). In addition, older adults with comorbid OSA and depression exhibited higher N1, N1%, and ArI values and lower N3 and N3% values than those with depression only (*p* < 0.05).

**Table 2:** Psychiatric characteristics and sleep architecture of older patients with depression according to the presence of OSA.

Variable	Depression with OSA (*n* = 52)	Depression without OSA (*n* = 108)	*p*
HAMD-24	26.2 ± 6.7	24.1 ± 7.7	0.086
TST (min), mean ± s.d.	444.5 ± 75.1	454.1 ± 67.4	0.419
SE (%), median (IQR)	83.0 (76.0, 90.0)	85.0 (78.0, 92.0)	0.153
SL (min), median (IQR)	22.5 (14.3, 29.0)	19.5 (11.0, 30.0)	0.394
WASO (min), median (IQR)	68.9 ± 28.4	60.7 ± 31.5	0.112
ArI, median (IQR)	19.6 ± 7.2	15.8 ± 6.9	0.002
REM, median (IQR)	54.5 (37, 69.8)	57.5 (41.3, 71.5)	0.504
REM%	12.0 (10.0, 14.0)	13.0 (10.0, 14.0)	0.513
N1, median (IQR)	51.0 (38.5, 71.0)	41.0 (30.0, 63.0)	0.026
N1%	11.0 (10.0, 13.8)	9.0 (7.0, 13.0)	<0.001
N2, median (IQR)	340.9 ± 36.5	346.0 ± 42.5	0.528
N2%	75.0 (74.0, 81.8)	76.0 (75.0, 77.0)	0.980
N3, median (IQR)	9.0 (3.3, 29.0)	14.0 (8.0, 38.0)	0.007
N3%	2.0 (1.0, 6.0)	3.0 (2.0, 7.8)	0.006

AHI, apnea-hypopnea index; HAMD-24, 24-item Hamilton Depression Rating Scale; IQR, interquartile range; TST, total sleep time; SE, sleep efficiency; SL, sleep latency, WASO, wake after sleep onset; ArI, arousal index; REM, rapid eye movement; REML, rapid eye movement latency; N1, non-rapid eye movement stage 1; N2, non-rapid eye movement stage 2; N3, non-rapid eye movement stage 3; N1%, N1/TST; N2%, N2/TST; N3%, N3/TST. Data summarized as mean ± s.d. were analyzed using the independent samples t-test. Data summarized as median (IQR) were analyzed using the Mann–Whitney U test.

### CTI and SII of older patients with depression according to the presence of OSA

As shown in Table 3, older adults with comorbid OSA and depression showed higher fasting glucose levels, triglyceride levels, CRP levels, triglyceride–glucose (TyG) index, and CTI than those with depression only (*p* < 0.05). It was also found that neutrophil counts, neutrophil-to-lymphocyte ratio (NLR), platelet-to-lymphocyte ratio (PLR), and SII were higher in older adults with comorbid OSA and depression than in those with depression only (*p* < 0.05).

**Table 3:** Biochemical examinations of older patients with depression according to the presence of OSA.

Variable	Depression with OSA (*n* = 52)	Depression without OSA (*n* = 108)	*p*
Fasting glucose (mg/dL), median (IQR)	108.0 (95.8, 119.3)	97.1 (86.6, 111.9)	< 0.001
Triglyceride (mg/dL), median (IQR)	142.0 (110.1, 156.6)	104.5 (78.4, 127.8)	< 0.001
CRP (mg/dL), median (IQR)	0.71 (0.51, 0.85)	0.54 (0.30, 0.70)	< 0.001
TyG index, median (IQR)	8.94 (8.60, 9.06)	8.52 (8.26, 8.79)	< 0.001
CTI, median (IQR)	8.78 (8.45, 8.87)	8.20 (7.94, 8.43)	< 0.001
Neutrophil (10^9^/L), median (IQR)	5.76 (4.70, 6.47)	4.04 (3.17, 5.49)	< 0.001
Platelet (10^9^/L), median (IQR)	263.5 (216.5, 287.0)	241.0 (202.3, 277.0)	0.058
Lymphocyte (10^9^/L), median (IQR)	1.76 (1.33, 2.26)	1.88 (1.50, 2.50)	0.122
NLR, median (IQR)	3.10 (2.17, 4.40)	2.03 (1.45, 3.11)	< 0.001
PLR, median (IQR)	136.2 (107.8, 175.6)	113.6 (101.0, 134.6)	0.003
SII, median (IQR)	670.0 (505.2, 1059.0)	465.8 (369.6, 678.4)	< 0.001

AHI, apnea-hypopnea index; IQR, interquartile range; CRP, C-reactive protein; TyG, triglyceride–glucose index; CTI, C-reactive protein–triglyceride–glucose index; NLR, neutrophil-to-lymphocyte ratio; PLR, platelet-to-lymphocyte ratio; SII, systemic immune-inflammation index. Data summarized as median (IQR) were analyzed using the Mann–Whitney U test.

### Logistic regression analysis

Logistic regression analysis was performed to evaluate which characteristics were jointly associated with the presence of OSA in depression, with sex, BMI, fasting glucose level, triglyceride level, CRP level, TyG index, CTI, neutrophil count, NLR, PLR, and SII selected as independent variables. The Hosmer–Lemeshow goodness-of-fit test (c^2^ = 3.73, df = 8, *p* = 0.881) indicated that the binary logistic regression model had an adequate fit. As shown in Table 4, in older adults with depression, logistic regression analysis demonstrated that male sex and higher CTI and SII values were correlated with the presence of OSA (Table 4, *p* < 0.05).

**Table 4:** Logistic regression analysis of risk factors for OSA in older patients with depression.

Variable	Coefficient (B)	*p*	OR	95% CI
Sex (male = 1; female = 0)	1.509	0.014	4.521	1.357–15.066
BMI (kg/m^2^)	0.081	0.293	1.084	0.933–1.259
Fasting glucose (mg/dL)	0.133	0.176	1.142	0.942–1.385
Triglyceride (mg/dL)	0.091	0.288	1.096	0.926–1.297
CRP (mg/dL)	1.660	0.486	5.262	1.049–23.864
TyG index	3.081	0.109	21.771	1.051–46.049
CTI	0.051	0.034	1.052	1.040–1.106
Neutrophil (10^9^/L)	0.525	0.333	1.690	0.585–4.887
Platelet (10^9^/L)	−0.005	0.735	0.995	0.969–1.022
NLR	0.409	0.606	1.664	1.140–4.146
PLR	0.017	0.339	1.017	0.982–1.054
SII	0.004	0.005	1.004	1.001–1.007

OR, odds ratio; 95% CI, 95% confidence interval; BMI, body mass index; CRP, C-reactive protein; TyG, triglyceride–glucose index; CTI, C-reactive protein–triglyceride–glucose index; NLR, neutrophil-to-lymphocyte ratio; PLR, platelet-to-lymphocyte ratio; SII, systemic immune-inflammation index.

## Discussion

This cross-sectional study of older adults with depression revealed a significant association between higher CTI and SII values and the presence of OSA. The association remained statistically significant following adjustment for confounders, suggesting that the CTI and SII may be associated with comorbid OSA and depression in older adults.

OSA is not only related to depressive symptoms but may also contribute to a reduced response to antidepressant pharmacotherapy, highlighting the importance of identifying specific risk factors for OSA (Titone et al., 2023). For older adults, the coexistence of OSA and depression may be associated with frailty development and an increased risk of cardiovascular disease (Xue et al., 2025; Zhao et al., 2023). In the general population, age, male sex, obesity, smoking, adenotonsillar hypertrophy, high blood pressure, and metabolic syndrome are considered risk factors for OSA (Salman et al., 2020; Giampa et al., 2023). The prevalence of OSA in older adults is significantly associated with age, increased BMI, obesity, cardiovascular diseases, diabetes, and daytime sleepiness (Ghavami et al., 2023). When we restricted the study population to older adults with depression, only male sex was identified as a risk factor for OSA. Although we also found a higher BMI in the comorbid group compared to the depression-only group, the association was not significant in the regression analysis. In older adults, being overweight was not found to be associated with a higher risk of all-cause mortality, and it seemed that older people with a BMI < 23.0 may require closer monitoring to address any modifiable causes of weight loss (Winter et al., 2014). In addition, BMI is also considered a risk factor for depression; however, in contrast, male individuals with abdominal obesity were less likely to experience depressive symptoms than male individuals without abdominal obesity (Luo et al., 2018). Since a greater proportion of men than women was observed in our sample of older adults with comorbid depression and OSA, and BMI is a risk factor for both depression and OSA, it may be reasonable that BMI was not significantly associated with OSA in older adults with depression after adjustment.

There were positive associations between OSA severity, lipid abnormalities, and diabetes mellitus (Gunduz et al., 2018; Yeghiazarians et al., 2021; Cass et al., 2013). The level of CRP remained significantly correlated with the AHI and desaturation index after controlling for covariates in patients with stable ischemic stroke and OSA (Chen et al., 2015). Data from 703 individuals with major depression demonstrated that the presence of metabolic syndrome and CRP > 7 mg/L were significant risk factors for moderate-to-severe OSA in major depression (Hein et al., 2017), indicating that lipid abnormalities and inflammation are more pronounced in this comorbid condition. The CTI, as an integrated marker based on the values of CRP, triglyceride, and glucose, has been used to simultaneously evaluate systemic inflammation and metabolic disturbances in chronic diseases, and its elevation in relation to OSA risk has been demonstrated in a large-scale population-based study (Yang and Su, 2025). Although the CTI was not reported in OSA, Popadic et al. (2022) found that patients with severe OSA had significantly higher levels of glucose, triglyceride, and CRP, which is consistent with the findings in our sample of older adults with depression. The SII has recently been explored as a novel inflammation marker, and its significant association with depression has been demonstrated in many patients with other physical diseases complicated by depression, such as diabetes mellitus (Ali et al., 2025). SII, NLR, and PLR parameters were found to be independently associated with OSA (Golen et al., 2024). Although no prior literature has reported an association between the SII and OSA in the context of depression, Wang et al. found that the SII mediated 6.1% of the obesity–depression relationship, compared to the NLR (5.2%) and SIRI (5.9%) (Wang et al., 2025), indicating that the SII may be indicative of metabolic disturbances in depression. As shown in our results, the SII was identified as a factor associated with the coexistence of OSA in older adults with depression. In addition to the CTI and SII, previous studies have identified brain asymmetry and circulating levels of interleukin-6 as potential factors associated with the coexistence of psychological disorders and OSA (Liao et al., 2024; Liao et al., 2025; Campos-Rodriguez et al., 2021), suggesting that identification of markers of this coexistence is important to uncover the pathogenesis of comorbid OSA and depression.

This study has several limitations. First, the sample size was relatively small for multivariable modeling, especially when examining correlated inflammatory and metabolic markers, necessitating external validation in larger cohorts. Second, although this study was adjusted for multiple confounders, there may be potential confounding factors. Third, the lack of serial sampling to monitor the dynamic status of the studied parameters may limit their interpretation and translation into clinical applications.

In conclusion, the findings obtained from this study demonstrate that elevated CTI and SII values are significantly associated with the coexistence of depression and OSA, reinforcing the involvement of inflammation in comorbid OSA and depression in older adults.

## Data Availability

The original contributions presented in the study are included in the article/supplementary material, further inquiries can be directed to the corresponding author.
